# The plant cell wall in the feeding sites of cyst nematodes

**DOI:** 10.3389/fpls.2014.00089

**Published:** 2014-03-19

**Authors:** Holger Bohlmann, Miroslaw Sobczak

**Affiliations:** ^1^Division of Plant Protection, Department of Crop Sciences, University of Natural Resources and Life SciencesVienna, Austria; ^2^Department of Botany, Warsaw University of Life SciencesWarsaw, Poland

**Keywords:** cell wall ingrowths, cell wall openings, cyst nematodes, feeding site, syncytium

## Abstract

Plant parasitic cyst nematodes (genera *Heterodera* and *Globodera*) are serious pests for many crops. They enter the host roots as migratory second stage juveniles (J2) and migrate intracellularly toward the vascular cylinder using their stylet and a set of cell wall degrading enzymes produced in the pharyngeal glands. They select an initial syncytial cell (ISC) within the vascular cylinder or inner cortex layers to induce the formation of a multicellular feeding site called a syncytium, which is the only source of nutrients for the parasite during its entire life. A syncytium can consist of more than hundred cells whose protoplasts are fused together through local cell wall dissolutions. While the nematode produces a cocktail of cell wall degrading and modifying enzymes during migration through the root, the cell wall degradations occurring during syncytium development are due to the plants own cell wall modifying and degrading proteins. The outer syncytial cell wall thickens to withstand the increasing osmotic pressure inside the syncytium. Furthermore, pronounced cell wall ingrowths can be formed on the outer syncytial wall at the interface with xylem vessels. They increase the surface of the symplast-apoplast interface, thus enhancing nutrient uptake into the syncytium. Processes of cell wall degradation, synthesis and modification in the syncytium are facilitated by a variety of plant proteins and enzymes including expansins, glucanases, pectate lyases and cellulose synthases, which are produced inside the syncytium or in cells surrounding the syncytium.

## Introduction

**Figure 1:**
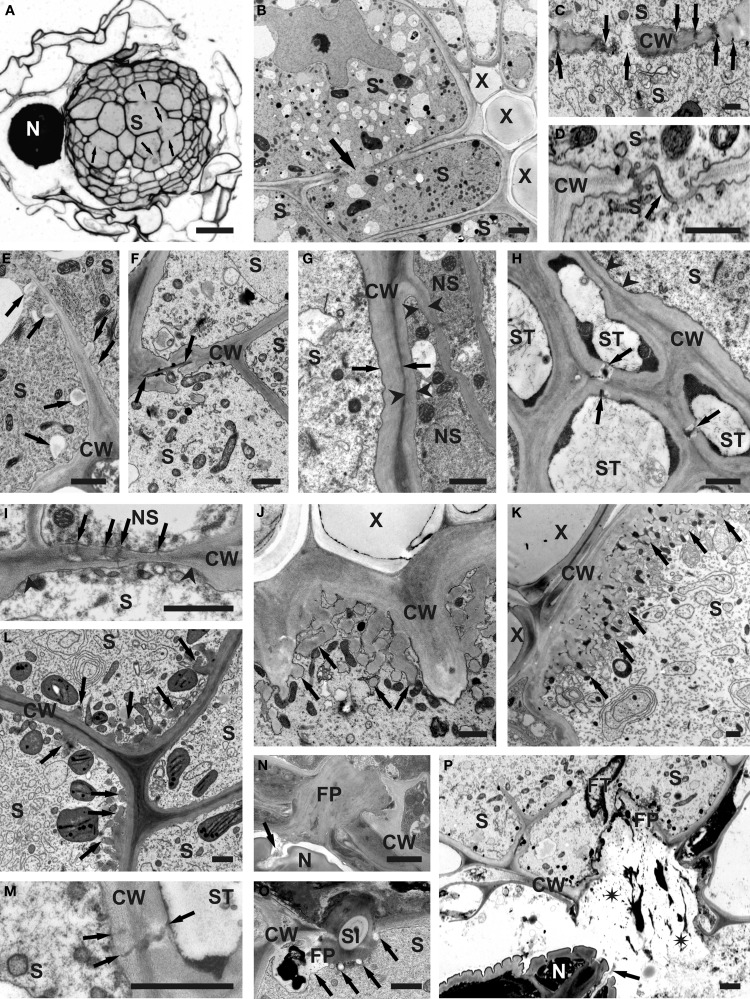
**Structural features of cell walls of syncytia induced by *H. schachtii* in *A. thaliana* roots**. **(A)** Anatomy of root containing syncytium. Arrows indicate cell wall openings. **(B)** Ultrastructure of root containing syncytium. Arrow indicates cell wall opening. **(C)** Cell wall openings formed by widening of plasmodesmata (arrows). **(D)** Cell wall openings formed by local dissolution of cell wall without involvement of plasmodesmata. Arrow indicates middle lamella covered with plasmalemma. **(E)** Paramural bodies (arrows) formed at extensively digested part of internal cell wall. **(F)** Casparian stripe (between arrows) covered with newly deposited cell wall in untypical syncytium induced in the endodermis. **(G)** Comparison of thickness of outer syncytial cell wall (between arrows) and cell wall of non-syncytial cells (between arrowheads). **(H)** Thin part of outer syncytial cell wall (arrowheads) facing sieve tube. Arrows indicate plasmodesmata between sieve tubes. **(I)** A group of plasmodesmata (arrows) at thin part of outer syncytial cell wall (between arrowheads) facing non-syncytial parenchymatous cell. **(J)** Single cell wall ingrowths (arrows) formed at syncytial wall facing vessels. **(K)** Well-developed system of cell wall ingrowths (arrows) formed at syncytial wall facing vessels. **(L)** Unusual localization of poorly developed cell wall ingrowths (arrows) on wall between syncytial elements. **(M)** Plasmodesmata (arrows) between syncytial element and sieve tube. **(N)** Feeding plug in syncytial cell wall. Secretions emanating from nematode amphids are marked with arrow. **(O)** Feeding plug with inserted cross-sectioned nematode stylet. Callose depositions are indicated with arrows. **(P)** Broken feeding plug in syncytial wall. Spilled syncytial cytoplasm is marked with asterisks. Arrow points to amphidal secretions. Light microscopy **(A)** and transmission electron microscopy micrograms **(B–P)** of syncytia at 2 **(E**,**O)**, 5 **(A**,**B**,**D**,**F,N,P)**, 10 **(G**,**H**,**I**,**J**,**M)**, and 13 **(C**,**K**,**L)** days post inoculation. CW, cell wall; FP, feeding plug; FT, feeding tube; N, nematode; NS, non-syncytial cell; S, syncytium; Sl, stylet; ST, sieve tube; X, xylem vessel. Bars = 20 μm **(A)** and 1 μm **(B–P)**.

Various species of plant parasitic nematodes attack the roots of crop plants, leading to serious agricultural losses, which have been estimated to be between US $ 125 and 157 billion per year (Chitwood, 2003; Abad et al., 2008). The sedentary cyst nematodes can be especially difficult to eradicate because they can survive in the soil for many years as dormant second stage juveniles (J2) in eggs protected by dead female bodies turned into cysts. Agriculturally the most important cyst nematode species are the potato cyst nematodes (*Globodera rostochiensis* and *G. pallida* with yellow/golden and white cysts, respectively) and the soybean cyst nematode (*Heterodera glycines*), which causes serious losses in soybean. Several other *Heterodera* species are found on cereals: *H. avenae* and *H. filipjevi* on oat, wheat and barley, and *H. zeae* on maize. The beet cyst nematode (*H. schachtii*) poses a serious problem for sugar beet production, but interestingly it is able to infect also species of the family *Brassicaceae* including *Arabidopsis thaliana* and this interaction is widely accepted as a model system in plant-nematode research (Sijmons et al., 1991). Upon stimulation by mostly unknown triggers, the migratory J2s hatch from the eggs and migrate toward roots. They enter an epidermal cell of host plant roots making numerous perforations in the cell wall with the aid of their stylet. Afterwards, they migrate intracellularly through the root cortical cells toward the vascular cylinder. J2s of *Globodera* sp. usually select an ISC among the inner cortex or the endodermal cells (Sobczak et al., 2005; Lozano-Torres et al., 2012) while *H. schachtii* selects an ISC among cambial or procambial cells inside the vascular cylinder (Golinowski et al., 1996; Sobczak et al., 1999). The cell wall of the ISC is pierced by the J2 with its stylet and secretions are injected directly into the plant cell cytoplasm through the hollow stylet (Wyss and Zunke, 1986; Wyss, 1992; Sobczak et al., 1999). These secretions are produced in the esophageal glands and although their nature and composition is still largely unknown, there is a common agreement that they contain effector proteins, which modify plant morphogenetic pathways thus facilitating the development of the syncytia (Hewezi and Baum, 2013). Neighboring cells fuse successively with the ISC through local cell wall dissolutions (Grundler et al., 1998) and thus a multinuclear syncytium composed of a large number of syncytial elements (=former root cells) is formed (Wyss and Grundler, 1992). The syncytium keeps expanding centripetally into the vascular cylinder by incorporation of pro-/cambial cells located between xylem and phloem bundles (Figures 1A,B; Golinowski et al., 1996; Sobczak et al., 1997) and acro- and basipetally along the root. The nematodes remain sedentary during their J2 and J3 developmental stages (Wyss, 1992). At the short-lasting J4 stage male nematodes cease food withdrawal and after the next molt they leave the roots as adult nematodes to search females for mating. The females continue to feed on syncytia during J4 and adult stages, and they never leave their feeding site. After insemination, they deposit hundreds of eggs mainly inside their body, which hardens to form the protective cyst (Wyss and Grundler, 1992).

The syncytium is the only source of nutrients for the cyst nematodes during their entire life cycle and thus it constitutes a severe sink in the plant because it has to be continuously “refilled” with nutrients necessary for the developing nematode. The nuclei of the syncytial elements enlarge and undergo endoreduplication (De Almeida Engler and Gheysen, 2013). Furthermore, also other ultrastructural features of the syncytial elements differ drastically from the ultrastructure of typical pro-/cambial cells. The large central vacuole is replaced by several small vacuoles and proliferating syncytial cytoplasm contains numerous plastids, mitochondria, ribosomes and structures of endoplasmic reticulum (Jones and Northcote, 1972; Bleve-Zacheo and Zacheo, 1987; Golinowski et al., 1996). In the course of these processes, the osmotic pressure in syncytia increases and becomes higher than in adjacent cells (Böckenhoff and Grundler, 1994). The ultrastructural features of syncytia implicate their high metabolic activity, which is confirmed by transcriptome analyses indicating up-regulation of genes related to high metabolic activity (Szakasits et al., 2009).

## The migratory phase

The invasion of the root and the development of the syncytium require both, destructive and constructive modifications of plant cell walls. It was originally believed that during invasion of the root, the cyst nematodes use only their stylet to make a series of punctures in the cell wall to break it solely with mechanical force of its stylet and lips (Wyss and Zunke, 1986; Wyss, 1992). However, several years ago it was evidenced that in addition to mechanical force also cell wall degrading enzymes are produced and secreted by the migratory J2s of cyst nematodes during their intracellular migration (Smant et al., 1998). The secreted cell wall degrading enzymes are produced in the two subventral gland cells, which are highly active in infective J2s, but atrophy when the nematodes become sedentary after induction of the ISC (Tytgat et al., 2002). In sedentary juveniles the dorsal gland cell becomes enlarged and more active thus it seems to be involved in further syncytium development and maintenance.

The plant cell wall is primarily composed of a variety of polysaccharides. Its exact composition is highly variable and depends on many factors such as the plant species, the cell type, the cell developmental stage as well as on external biotic and abiotic factors (Keegstra, 2010). Its main component is cellulose, a β-1,4-linked glucan, which forms crystalline microfibrils cross-linked by hemicelluloses: xyloglucan, glucomannan, xylan, and mixed-linkage glucans (Scheller and Ulvskov, 2010). These are embedded in a complex polysaccharide matrix of pectin: homogalacturonan and rhamnogalacturonan I and II (Harholt et al., 2010). In addition, cell walls can be lignified (Li and Chapple, 2010) and they can also contain a variety of different proteins and glycoproteins (Rose and Lee, 2010).

The plant cell wall is a barrier for many pathogens and various cell wall polysaccharide degrading enzymes have been described for many different pathogens (for review see Hematy et al., 2009). Cyst nematodes also produce a variety of cell wall polysaccharide degrading enzymes during migration through the root. However, in contrast to fungal pathogens, which usually secrete first pectin degrading enzymes, there is no indication that nematode secreted cell wall degrading enzymes are released in chronological or sequential order. Rather, it seems that a cocktail of various polysaccharide degrading enzymes is produced in the nematode esophageal glands and secreted through the stylet orifice (for review see Davis et al., 2011).

Two groups of pectin degrading enzymes are produced by plant parasitic nematodes, which are specifically active on unmethylated polysaccharides: polygalacturonases and pectate lyases (see Table 1 for the genes/proteins from nematodes mentioned in this review). The former cleave bonds between galacturonic acids in (unmethylated) homogalaturonan and they were found in secretions of root knot nematodes (Jaubert et al., 2002), but not in cyst nematodes. The latter have been identified in several cyst nematode species, including *H. glycines* (De Boer et al., 2002), *H. schachtii* (Vanholme et al., 2007), and *G. rostochiensis* (Popeijus et al., 2000; Kudla et al., 2007). Down-regulation of one of the two pectate lyase genes present in the *H. schachtii* genome by RNAi resulted in a lower number of infecting juveniles, thus clearly demonstrating the importance of pectate lyases for root invasion and successive migration of the nematode inside the host root (Vanholme et al., 2007).

**Table 1:** **Nematode proteins acting on plant cell walls**.

**Gene/Protein**	**Nematode**	**Class**	**Function**	**References**
*Hg-pel-1*	*H. glycines*	Pectate lyase		De Boer et al., 2002
*Hspel1*	*H. schachtii*	Pectate lyase		Vanholme et al., 2007
*Hspel2*			Silencing results in low infection efficiency	
Gr-PEL1	*G. rostochiensis*	Pectate lyase		Popeijus et al., 2000
Gr-PEL2			Transient expression in *Nicotiana benthamiana* resulted in severe malformations of infiltrated tissues	Kudla et al., 2007
GR-ENG-1	*G. rostochiensis*	Cellulase	Hydrolysis of carboxymethylcellulose	Smant et al., 1998
GR-ENG-2	*G. rostochiensis*			
HG-ENG-1	*H. glycines*			
HG-ENG-2	*H. glycines*			
GR-ENG-1/2/3/4	*G. rostochiensis*	Cellulase	Targeting the mRNAs of secreted cellulases with dsRNA resulted in lower nematode invasion	Chen et al., 2005
Gr-EXPB1	*G. rostochiensis*	Expansin	Cell-wall-extension activity	Qin et al., 2004; Kudla et al., 2005
Hs CBP	*H. schachtii*	Cellulose binding protein	Interacts with a plant pectin methylesterase (PME3) to aid cyst nematode parasitism	Hewezi et al., 2008

Genes are shown in italic for those cases where the proteins have not been reported.

Cellulases (β-1,4-endoglucanases) found in *G. rostochiensis* and *H. glycines* were the first cell wall degrading enzymes encoded by animal genomes that were discovered, which was surprising at that time because it was a general thought that animals are unable to produce these enzymes themselves but depended on symbiotic microorganisms for that purpose (Smant et al., 1998). These proteins are produced in the subventral gland cells and they are secreted during nematode migration as indicated by their immunolocalization in plant tissues (Wang et al., 1999). Secreted cellulases can be involved in the softening and weakening of the cellulose network in plant cell walls during the root invasion and intracellular migration phases making mechanical cell wall rupture easier. Their importance for cyst nematode parasitism was demonstrated by RNAi silencing of their expression in *G. rostochiensis*. Incubation of invasive J2s with dsRNA targeting the mRNA of secreted cellulases resulted in fewer nematodes that could invade and induce syncytia in the host roots (Chen et al., 2005).

Genomes of sedentary plant parasitic nematodes encode also expansins (Qin et al., 2004; Kudla et al., 2005). Expansins are widespread in plants, but had not been found outside the plant kingdom before. They have no enzymatic activity but function by loosening or weakening of the non-covalent bonds between cellulose microfibrills and associated hemicelluloses, thus making the cell wall more accessible for cell wall degrading enzymes, and more responsive to turgor-driven plant cell growth (McQueen-Mason and Cosgrove, 1994). Loosening of the plant cell walls by expansins produced by migratory juveniles would result in better accessibility for nematode secreted cell wall degrading enzymes and could also facilitate mechanical disruption with the aid of the nematode stylet and lips (Qin et al., 2004).

We can conclude that there is a bulk of evidence indicating that during root invasion and migration through the root tissues the cyst nematodes use mechanical force in combination with a sophisticated set of enzymes that soften the rigidity of plant cell walls through polysaccharide degrading enzymes and cell wall modifying proteins. Once the J2 has selected an ISC, it becomes sedentary and changes its strategy. Now, the dorsal gland cell becomes more active, indicating that the nematode is producing a different set of parasitism-related proteins (effectors). The ISC develops into a syncytium via hypertrophy of affected plant cells and formation of partial cell wall dissolutions between them leading to the formation of a confluent protoplast inside the entire feeding site. This again requires activities of cell wall degrading enzymes and modifying proteins such as expansins, but now they are apparently produced by the plant, not by the nematode. There have been no reports that cell wall degrading enzymes produced by the nematode are secreted into the syncytium, although, this cannot be excluded. Instead, the sedentary plant parasitic nematodes have developed a sophisticated system of effectors released into the feeding site, which allow them now to avoid plant defense responses and to “persuade” the plant to work for parasite benefits (Quentin et al., 2013).

## The sedentary phase

The incorporation of root cells into syncytia requires a local dissolution of cell walls (Figures 1A,B; Grundler et al., 1998) and it has been shown that several plant proteins are involved in this process, including expansins, cellulases, and pectinases. The expression of these proteins has to be tightly regulated and some of the genes encoding expansins and cell wall degrading enzymes are specifically temporally and spatially regulated in syncytia or in the surrounding cells (Wieczorek et al., 2006, 2008; Szakasits et al., 2009). Most of the knowledge concerning cell wall modifications has been gained from the research on the interaction between *H. schachtii* and *A. thaliana* roots; therefore we will concentrate on this interaction (see Table 2 for the genes mentioned in this review). Further information on other host plants and other cyst (and root knot nematodes) can be found in a review by Sobczak et al. (2011).

**Table 2:** **Plant genes involved in syncytium formation**.

**Gene**	**Name**	**Class**	**Function/Expression**	**References**
*At2g37640*	AtEXPA3	Expansin	Up-regulated in syncytia, loosening of cell walls	Wieczorek et al., 2006
*At3g55500*	AtEXPA16			
*At1g70710*	AtCel1	Cellulase	Expressed in young syncytia. Implicated in cell wall softening during early stages of syncytium development	Mitchum et al., 2004
				Wieczorek et al., 2008
*At1g02800*	AtCel2	Cellulase	Degradation of cell walls. Decreased numbers of females develops on roots of T-DNA mutants	Wieczorek et al., 2008
*At4g24260*	AtKor3			
*At3g14310*	Pme3	Pectin methylesterase	Interacts with a cellulose binding protein secreted by *H. schachtii* during early phase of syncytium development	Hewezi et al., 2008
*At3g05910*	PAE (DiDi 9C-12)	Pectin acetylesterase	Up-regulated in and around young expanding syncytia induced by *H. schachtii*	Vercauteren et al., 2002
*At3g27400*	PLL18	Pectate lyase-like	Up-regulated in syncytia. Important for proper development of the cyst nematode *H. schachtii*	Wieczorek et al., unpublished
*At4g24780*	PLL19			
*At3g54920*	PMR6	Pectate lyase-like	Up-regulated in syncytia. Known also as powdery mildew susceptibility gene	Vogel et al., 2002
				Szakasits et al., 2009
*At1g14520*	MIOX1	Myo-inositol oxygenase	Strong expression in syncytia. Important for syncytium and nematode development, probably due to removal of excess myo-inositol to decrease the level of galactinol	Siddique et al., 2009, 2014
*At2g19800*	MIOX2			
*At4g26260*	MIOX4			
*At5g56640*	MIOX5			
*At5g39320*	UGD1	UDP-glucose dehydrogenase	Expressed in syncytia	Siddique et al., 2012
*At3g29360*	UGD2		Expressed in syncytia. In mutant plants cell wall ingrowths are not formed in syncytia	
*At5g15490*	UGD3			
*At1g26570*	UGD4		Expressed in syncytia	

The formation of syncytia requires the fusion of root cell protoplasts by partial cell wall dissolution (Figures 1A,B; Grundler et al., 1998). During early stages of syncytium development it is achieved by widening of plasmodesmata between the ISC and neighboring cells (Figure 1C; see also Figures 3, 4 in Grundler et al., 1998), while at later stages cell wall openings are mostly formed by dissolution of cell wall fragments without the involvement of plasmodesmata (Figure 1D; see also Figures 8, 9 in Grundler et al., 1998). However, when a group of cells is incorporated into a syncytium, cell wall dissolutions can be formed among distal cells by widening of plasmodesmata. Expansins are apparently helper-proteins in this process. As it has been mentioned, expansins do not have enzymatic activity, but they are involved in all processes that require the expansion of plant cells or the degradation of cell walls. They act by weakening the hydrogen bonds between cell wall polysaccharides (McQueen-Mason and Cosgrove, 1994). The *A. thaliana* genome contains 26 genes coding for α-expansins and 5 genes coding for β-expansins, which are differentially regulated in different plant organs and in feeding sites of the cyst nematode *H. schachtii* (Wieczorek et al., 2006). Some expansin genes are expressed in roots and syncytia while some others are expressed in roots but down-regulated or silenced in syncytia. Genes, such as *AtEXPA3* (*At2g37640*) and *AtEXPA16* (*At3g55500*), which are expressed in shoots but not in roots in uninfected plants, are specifically up-regulated in syncytia, indicating that they are important for syncytium development.

When the texture of plant cell walls is loosened by expansins, it becomes more accessible for a variety of plant derived enzymes, which are involved in cell wall degradation. These enzymes include cellulases (endo-1,4-β-glucanases) and different pectin degrading enzymes. Goellner et al. (2001) were the first who showed that plant cellulase genes are expressed in tobacco roots infected with *G. tabacum*. A detailed analysis of the cellulase gene expression pattern in *Arabidopsis* roots infected with *H. schachtii* showed a complex picture resembling the expression pattern of expansin genes (Wieczorek et al., 2008). The *A. thaliana* genome contains 25 genes annotated as cellulases. Seven of them were up-regulated and seven were down-regulated in syncytia. GeneChip analysis indicated the *AtCel1* gene (*At1g70710*) as being up-regulated in syncytia at 5 days post inoculation (dpi), while GUS analysis did not detect expression of the *AtCel1* promoter in 10 dpi syncytia (Mitchum et al., 2004). The reason for these divergent results might be that this gene is apparently expressed only in young syncytia. Other up-regulated genes are *AtCel2* (*At1g02800*) and *AtKor3* (*At4g24260*). Their importance for feeding site and nematode development was demonstrated with mutant studies. Only about half of the number of females developed on roots of *AtCel2* or *AtKor3* mutants in comparison to wild type plants (Wieczorek et al., 2008).

Another important group of enzymes involved in degradation of cell walls during the development of feeding sites induced by the cyst nematodes are enzymes taking part in degradation and modifications of pectins. These enzymes are encoded by large gene families in the *A. thaliana* genome (see supplementary tables). There are 66 genes coding for polygalacturonases (Kim et al., 2006), 66 genes encoding pectin methylesterases (Louvet et al., 2006), 12 genes encoding pectin acetylesterases (Gou et al., 2012), 26 genes annotated as pectate lyases or pectate lyase-like genes (Palusa et al., 2007; Sun and van Nocker, 2010) and 67 genes coding for pectin lyases (Cao, 2012). However, it has to be kept in mind that this classification is based at the moment on *in silico* analyses and that the enzymatic activity of most proteins has not been tested *in vivo* or *in vitro*. During syncytium development the action of these enzymes seems to be of special importance to allow intrusive growth of enlarging syncytial elements among surrounding parenchymatous cells (Figures 1A,B). Their action seems also necessary for proper formation of cell wall openings when neighboring protoplasts become separated by middle lamella only (Figure 1D; see also Grundler et al., 1998). It has to be digested to allow formation of confluent syncytial cytoplasm. However, during enlargement of syncytial elements the exposed middle lamella might also be torn apart mechanically. When the cell wall openings expand numerous paramural bodies of different sizes are formed (Figure 1E; see also Figure 11 in Golinowski et al., 1996).

Very little is currently known about the expression of genes coding for pectin-degrading enzymes in feeding sites of *H. schachtii*. The transcriptome analysis conducted by Szakasits et al. (2009) showed that among 67 genes coding for pectin lyases four were up-regulated and four were down-regulated in syncytia as compared to control root segments (a false discovery rate at <5% was used). None of these genes was expressed at a high level, indicating that pectin lyases might be less important for the degradation of cell walls leading to incorporation of new root cells into the growing syncytium than pectate lyases (discussed below).

While pectin lyases are able to hydrolyze methylated pectin, polygalacturonases and pectate lyases are unable to degrade methylated pectin. These enzymes can only work once the pectin is demethylated or deacetylated by pectin methylesterases or acetylesterases. Out of the 66 genes encoding pectin methylesterases three were up-regulated and 10 were down-regulated in syncytia induced by *H. schachtii* according to data reported by Szakasits et al. (2009). The protein encoded by *Pme3* (*At3g14310*) interacts with a cellulose binding protein secreted by *H. schachtii* during early phases of syncytium development. Transgenic plants overexpressing this pectin methylesterase were more susceptible to infection with *H. schachtii* while a knockout mutant showed the opposite effect, indicating the importance of Pme3 for nematode parasitism (Hewezi et al., 2008).

Among 12 putative pectin acetylesterase genes, two were down-regulated and three were up-regulated in syncytia according to data reported by Szakasits et al. (2009). One of the latter genes (*At3g05910*) was up-regulated both in young giant cells induced by the root-knot nematode *M. incognita* as well as in syncytia induced by *H. schachtii* (Vercauteren et al., 2002). Deacetylation and demethylation makes the pectin accessible for degradation by polygalacturonases and pectate lyases. Four out of the 66 genes encoding putative polygalacturonases in the *A. thaliana* genome were slightly up-regulated while five were down-regulated in syncytia induced by *H. schachtii*. Additionally, there are also 26 genes in the *A. thaliana* genome coding for pectate lyase-like (PLL) enzymes (Palusa et al., 2007). Six of them were up-regulated and four were down-regulated in syncytia when compared to uninfected *Arabidopsis* roots according to data reported by Szakasits et al. (2009). These results, with all due precautions, indicate that pectate lyases might be more important for the degradation of cell walls in syncytia than polygalacturonases and recent work by Wieczorek et al. (pers. comm.) supports this assumption. They studied two PLL genes (*PLL18*; *At3g27400* and *PLL19*; *At4g24780*), which are up-regulated in syncytia. Analyses of T-DNA mutants showed that both genes are important for proper development of the cyst nematode *H. schachtii*, but not for the root knot nematode *M. incognita*. It should also be stressed that one pectate lyase-like gene (*PMR6*; *At3g54920*), which is also up-regulated in syncytia was identified as a powdery mildew susceptibility gene in *A. thaliana* (Vogel et al., 2002). With a few exceptions, our information about the role of the different pectin modifying/degrading enzymes for syncytium development is still very limited and fragmentary.

It seems that plant parasitic nematodes do not have enzymes degrading suberin as well as they are not able to modify expression of plant genes coding for enzymes degrading it. The juveniles of the root knot nematode *M. incognita* invade plant roots close to the root tip. They enter the root and migrate toward the vascular cylinder intercellularly dissolving the middle lamellae. When they reach the endodermis with developed Casparian stripes (narrow bands of suberinized cell wall) they turn first toward the root tip and then turn again toward the center of the vascular cylinder just above the root tip meristem where the Casparian stripes are not yet formed (Wyss et al., 1992). In the case of syncytia induced by *Globodera* sp., which are induced in the root cortex, the cell wall openings were never formed at parts of endodermal cell walls with Casparian stripes. In general, suberinized Casparian stripes are not a problem for *Heterodera* sp., which usually induce syncytia among cells of the vascular cylinder and migrate through the endodermis intracellularly. However, in a very few cases, syncytia induced in young parts of *A. thaliana* roots incorporated also endodermal cells with developed Casparian stripes (Figure 1F). In such cases the suberinized part of the plant cell wall remained undigested and was covered by thick layers of newly deposited cell wall material. It indicates that the cyst nematodes are unable to regulate the expression of plant suberin-digesting enzymes and do not produce these enzymes themselves.

Concomitantly with processes of cell wall degradation, the development of syncytia also requires the synthesis of new cell wall materials. The outer syncytial cell wall is thickened (Figures 1B,G; see also: Figures 25, 26 in Sobczak et al., 1997; Figure 10 in Golinowski et al., 1996) to withstand and counteract the high osmotic pressure inside the syncytia, which can reach 10,000 hPa in syncytia induced by *H. schachtii* in *A. thaliana* roots (Böckenhoff and Grundler, 1994). While the outer cell wall is generally thickened (Figure 1G; see also: Figures 25, 26 in Sobczak et al., 1997; Figure 10 in Golinowski et al., 1996), those parts of the syncytial wall that face sieve tubes (Figure 1H; see also: Figure 5 in Grundler et al., 1998; Figure 19.1A in Sobczak et al., 2011) or cells being incorporated into syncytia (Figure 1I; see also Figure 19.1B in Sobczak et al., 2011) often remain thin while the sieve tube wall becomes thickened. This might favor the uptake of solutes into the syncytium. In syncytia associated with female nematodes cell wall ingrowths are developed at the interface with xylem vessels (Jones and Northcote, 1972; Golinowski et al., 1996). The first cell wall ingrowths are formed as dispersed finger-like protrusions (Figure 1J; see also Figure 5E in Siddique et al., 2012) in 5–7 dpi syncytia. Later they ramify and expand forming pronounced cell wall labyrinths imprisoning mitochondria and vesicles (Figure 1K; see also: Figures 5–7, 27 in Jones and Northcote, 1972; Figure 20 in Golinowski et al., 1996). In syncytia associated with male juveniles, which have only two developmental stages of active food uptake and thus lower nutritional demands than female juveniles (Müller et al., 1981), and in syncytia induced in plants grown under adverse conditions the disperse and single cell wall ingrowths are formed at different locations, for instance between syncytial elements or on the outer syncytial wall (Figure 1L; see also Figures 28, 29 in Sobczak et al., 1997). Cell wall ingrowths are a characteristic feature of transfer cells differentiating at various locations in plant organs. They are ubiquitous in plants and are also found in fungi and algae (Talbot et al., 2002; Offler et al., 2003). It has been proposed that the role of cell wall ingrowths is to increase the surface of the interface between symplast and apoplast thus allowing a higher exchange of solutes. As the cell wall ingrowths in syncytia are formed at the xylem interface it might be assumed that they enhance the uptake of water from the vessels because it was calculated that female juveniles withdraw four times the syncytium volume daily (Müller et al., 1981).

Thickening of the outer syncytial wall begins soon after syncytium induction (Sobczak et al., 1999). It usually leads to occlusion of plasmodesmata, if they have not been widened and turned into cell wall openings (Figure 1C; see also Figures 3, 4 in Grundler et al., 1998). Thus, for a long time it was generally accepted that syncytia are symplasmically isolated. However, recently several papers have been published providing indirect evidence that plasmodesmata are formed *de novo* in outer syncytial cell walls and that the syncytium is uploaded simplasmically (Hoth et al., 2005; Hofmann and Grundler, 2006; Hofmann et al., 2007). However, there is no ultrastructural support for functional plasmodesmata in the outer cell wall of syncytia. Plasmodesmata in the outer syncytial wall can be found only occasionally in cell walls facing sieve tubes (Figure 1M; see also: Figure 5 in Grundler et al., 1998; Figures 19.2C,D in Sobczak et al., 2011) or parenchymatous cells being incorporated into syncytia (Figure 1I; see also Figures 19.2E,F in Sobczak et al., 2011). If present, they usually seem to be occluded by deposited cell wall material from the syncytial side. However, plasmodesmata are present abundantly between parenchymatous cells surrounding syncytia (see also Figure 19.3A in Sobczak et al., 2011).

The feeding plug is another structure characteristic for syncytial cell walls, which is not found in giant cells induced by root knot nematodes. It is embedded into the outer syncytial cell wall at the place where the nematode stylet is inserted into the syncytium (Figures 1N,O; see also Figures 5, 6, 8 in Sobczak et al., 1999). Presumably, this structure is important as a seal preventing leakage of the syncytial cytoplasm along the inserted stylet (Figure 1O; see also Figures 6, 8 in Sobczak et al., 1999). It must be soft enough to allow stylet insertion, but also strong enough to prevent cell wall rupture when the cell wall is punctured with the nematode stylet. If the stylet is inserted outside the feeding plug it can lead to cell wall rupture and syncytium collapse, although a callose-based repair mechanism is activated (Figure 1P). The origin and chemical composition of the feeding plug remain obscure. A small feeding plug is observed in syncytia already several hours after ISC selection. It enlarges and often becomes multipartite during syncytium development. The feeding plug is probably mostly composed of callose that is abundantly deposited around the tip (except the canal orifice) of the inserted stylet (Figure 1O; see also Figure 8 in Sobczak et al., 1999). The stylet is withdrawn in every feeding cycle, approximately every 3–4 h (Wyss, 1992), which could lead to leakage from the syncytium. This is prevented by the callose, which is pulled into the opening in the cell wall. However, active formation of the feeding plug by the secretions originating from nematode amphids cannot be ruled out (Figures 1N,P; see also: Figure 3 in Endo, 1978; Figure 7 in Sobczak et al., 1999).

The major precursor for synthesis of cell wall polysaccharides in *Arabidopsis* is UDP-glucuronic acid, which can be produced through two different pathways. Under normal growth conditions UDP-glucose dehydrogenase (UGD) supplies the majority of UDP-glucuronic acid from UDP-glucose. A second pathway involves the enzyme myo-inositol oxygenase (MIOX) converting myo-inositol to D-glucuronic acid, which is thereafter converted to D-glucuronic acid-1 phosphate by glucuronokinase, and finally to UDP-glucuronic acid by UDP-sugar pyrophosphorylase (Kanter et al., 2005; Klinghammer and Tenhaken, 2007). Myo-inositol is produced from glucose 6-phosphate through the rate-limiting conversion to myo-inositol-3-phosphate, which is catalyzed by the enzyme myo-inositol-phosphate synthase (MIPS). Myo-inositol-3-phosphate is then dephosphorylated to myo-inositol by myo-inositol monophosphatases (Loewus and Murthy, 2000).

MIOX is encoded by 4 genes in the *A. thaliana* genome. *MIOX1* and *MIOX2* are expressed preferentially in seedlings while *MIOX4* and *MIOX5* are highly expressed in pollen grains (Kanter et al., 2005). Expression of all 4 *MIOX* genes is strongly elevated in syncytia induced by *H. schachtii* (Siddique et al., 2009). Double mutants of the four *MIOX* genes supported development of a significantly reduced number of *H. schachtii* females indicating the importance of this pathway for the proper development of syncytia and associated nematodes (Siddique et al., 2009). However, no differences could be detected between *miox* double mutants, a quadruple mutant and wild type plants in biochemical composition and ultrastructure of syncytial cell walls (Siddique et al., 2009, 2014). The importance of the MIOX pathway for syncytium development is probably not in the production of cell wall precursors, but rather in the removal of excess myo-inositol from syncytia. A high level of myo-inositol would lead to a higher level of galactinol and there is evidence that the high galactinol level leads to decreased susceptibility of *miox* mutants against *H. schachtii* (Siddique et al., 2014).

There is also a small gene family in the *A. thaliana* genome consisting of four genes that encode UGD (*UGD1*, *UGD2*, *UGD3*, and *UGD4*) (Klinghammer and Tenhaken, 2007). *UGD1* is only weakly expressed in roots while *UGD2*, *UGD3*, and *UGD4* are expressed at high levels in roots. We have studied the expression pattern of these genes in syncytia using promoter::GUS lines (Siddique et al., 2012). All four genes are expressed in syncytia; *UGD2* and *UGD3* as early as 1 dpi while expression of *UGD1* and *UGD4* was detected starting at 2 dpi. A mutant analysis revealed that single *UGD* mutants (Δ *ugd2* and Δ *ugd3*) support development of fewer and smaller females, which feed from smaller syncytia as compared to wild type plants. The double mutant Δ Δ *ugd23* had an even stronger effect than the single mutants. Ultrastructural examination of syncytia induced in the ΔΔ*ugd23* double mutant revealed that syncytia usually contained an electron translucent cytoplasm with degenerating organelles and cell wall ingrowths were absent. These results showed that *UGD2* and *UGD3* are needed for the formation of cell wall ingrowths in syncytia (Siddique et al., 2012).

Knowledge concerning the chemical composition of syncytial cell walls is very limited. The basic reason for this situation is the general difficulty to obtain a sufficient amount of purified material for biochemical analyses. Therefore, immunological methods are the only reasonable method to study the chemical composition of syncytial cell walls. Using monoclonal antibodies, which target specific components of plant cell wall polysaccharides, cell wall composition was examined in syncytia induced by *H. schachtii* in *A. thaliana* roots (Davies et al., 2012). These analyses demonstrated the presence of cellulose and hemicelluloses such as xyloglucan and heteromannan. Xylan was not detected, indicating that syncytia are devoid of secondary cell walls although outer cell walls of syncytia are strongly thickened. The pectin in syncytial walls appeared to be heavily methyl-esterified, which could explain the rather low expression of pectin lyases in syncytia as these enzymes are able to degrade only methylated pectin, but not de-esterified one. There is at the moment no explanation why the higher expression of *AtPme3* was positively correlated with susceptibility because a higher expression of pectin methylesterases might not only result in an increased degradation of cell walls between syncytial elements, which might favor syncytium development, but also should lead to an enhanced degradation of the outer syncytial cell wall and thus have detrimental effects on the syncytium. One might therefore speculate that there are certain chemical differences between inner and outer cell walls of syncytia, however, the inner cell walls (those between syncytial elements) are also not completely degraded but only locally dissolved. An explanation for this phenomenon could be that cell wall degrading enzymes produced in syncytia are very specifically targeted to only those parts of the cell walls between syncytial elements that are scheduled to be degraded or that the outer cell walls of syncytia are protected through the deposition of enzyme inhibitors.

Examination of the chemical composition of the outer syncytial cell wall (Davies et al., 2012) indicates that it is on the one hand strong enough to withstand the high internal turgor pressure of the syncytium and on the other hand it remains flexible (Davies et al., 2012). This flexibility of outer syncytial walls seems to be especially important when we consider that feeding of the cyst nematodes occurs in cycles (Wyss and Zunke, 1986; Wyss, 1992). During each feeding cycle a substantial part of the syncytium protoplast is withdrawn by the nematode. It has been calculated that an adult female of *H. schachtii* can withdraw four times the volume of the syncytium through its stylet per day (Müller et al., 1981). The flexibility of the outer syncytial cell wall allows the syncytium to contract during the active food withdrawal stage when the nematode feeds and takes up a large part of the syncytial volume with solutes and to expand when no food is withdrawn and the syncytium is “refilled” from conductive elements.

### Conflict of interest statement

The authors declare that the research was conducted in the absence of any commercial or financial relationships that could be construed as a potential conflict of interest.
