# Supplementary material for: The plant cell wall in the feeding sites of cyst nematodes
Source: Front Plant Sci. 2014 Mar 19;5:89. doi: 10.3389/fpls.2014.00089 (PMC3958752; doi:10.3389/fpls.2014.00089)
Supplement: Supplementary file 1 [file DataSheet1.DOCX]

**Supplementary Material**

The tables show different Arabidopsis gene families for cell wall degrading enzymes. The data for expression of these genes in syncytia or control root segments are from Szakasits et al. (2009). In addition, up- or down-regulation in syncytia is indicated, also according to Szakasits et al (2009). – indicates that the gene is not covered by the ATH1 GeneChip.

**Arabidopsis pectin lyases**

Cao, J. (2012). The pectin lyases in *Arabidopsis thaliana*: evolution, selection and expression profiles. *PLoS One* 7**,** e46944.

| Gene | Syncytium | Root | Regulation |
| --- | --- | --- | --- |
|  |  |  |  |
| At1g02460 | 4.0 | 4.7 | down |
| At1g02790 | 3.0 | 3.2 |  |
| At1g05650 | - | - |  |
| At1g05660 | - | - |  |
| At1g10640 | 2.7 | 2.9 |  |
| At1g17150 | 2.9 | 2.9 |  |
| At1g19170 | 6.1 | 6.1 |  |
| At1g23460 | - | - |  |
| At1g43080 | - | - |  |
| At1g43090 | - | - |  |
| At1g43100 | - | - |  |
| At1g48100 | 2.8 | 2.5 |  |
| At1g56710 | 2.7 | 2.7 |  |
| At1g60590 | 2.6 | 2.7 |  |
| At1g65570 | 2.6 | 3.0 |  |
| At1g70500 | - | - |  |
| At1g78400 | 2.4 | 2.4 |  |
| At1g80140 | 3.1 | 3.1 |  |
| At1g80170 | 5.5 | 4.4 | up |
| At2g15450 | - | - |  |
| At2g15460 | - | - |  |
| At2g15470 | - | - |  |
| At2g23900 | 2.1 | 2.2 |  |
| At2g26620 | 2.4 | 2.6 |  |
| At2g33160 | 2.7 | 2.7 |  |
| At2g40310 | 2.9 | 3.0 |  |
| At2g41850 | 3.2 | 3.5 |  |
| At2g43860 | 3.3 | 3.4 |  |
| At2g43870 | 2.6 | 2.9 |  |
| At2g43880 | 2.4 | 2.6 |  |
| At2g43890 | 2.9 | 2.9 |  |
| At3g06770 | 4.3 | 5.2 |  |
| At3g07820 | 2.4 | 2.5 |  |
| At3g07830 | 3.2 | 3.4 |  |
| At3g07840 | 2.4 | 2.5 |  |
| At3g07850 | - | - |  |
| At3g07970 | 2.7 | 3.0 |  |
| At3g14040 | - | - |  |
| At3g15720 | 3.2 | 3.3 |  |
| At3g16850 | 5.1 | 5.6 |  |
| At3g26610 | 3.3 | 5.6 | down |
| At3g42950 | 3.5 | 3.9 |  |
| At3g48950 | 3.8 | 3.8 |  |
| At3g57510 | 3.4 | 3.5 |  |
| At3g57790 | 5.6 | 9.1 | down |
| At3g59850 | 2.6 | 2.7 |  |
| At3g61490 | 5.6 | 5.1 | up |
| At3g62110 | 4.8 | 4.3 |  |
| At4g01890 | 3.9 | 4.7 |  |
| At4g13760 | 2.3 | 2.4 |  |
| At4g18180 | 3.0 | 3.3 |  |
| At4g23500 | - | - |  |
| At4g23820 | 5.6 | 3.9 | up |
| At4g32370 | - | - |  |
| At4g32375 | 2.9 | 3.0 |  |
| At4g32380 | - | - |  |
| At4g33440 | 5.0 | 4.6 |  |
| At4g35670 | 2.7 | 2.9 |  |
| At5g14650 | 3.0 | 3.6 | down |
| At5g17200 | 2.9 | 2.9 |  |
| At5g27530 | 3.8 | 4.0 |  |
| At5g39910 | 2.5 | 2.6 |  |
| At5g41870 | 4.2 | 3.6 | up |
| At5g44830 | 3.0 | 2.9 |  |
| At5g44840 | 3.0 | 2.9 |  |
| At5g48140 | 2.2 | 2.4 |  |
| At5g49215 | - | - |  |

**Arabidopsis pectate lyases like (PLL)**

Palusa, S.P., Golovkin,M., Shin, S.-B., Richardson, D.N. and Reddy, A.S.N. (2007). Organ-specific, developmental, hormonal and stress regulation of expression of putative pectate lyase genes in Arabidopsis. *New Phytol* 174, 537-50.

|  | Syncytium | Root | Regulation | Other names |
| --- | --- | --- | --- | --- |
|  |  |  |  |  |
| At5g63180 | 2.4 | 2.7 |  | PLL15 |
| At1g67750 | 6.1 | 3.3 | up | PLL16 |
| At3g27400 | 9.5 | 2.2 | up | PLL18 |
| At4g13710 | 3.6 | 3.5 |  | PLL25 |
| At3g24230 | 2.2 | 2.4 |  | PLL24 |
| At1g04680 | 4.1 | 3.4 |  | PLL26 |
| At4g13210 | 2.5 | 2.6 |  | PLL23 |
| At3g24670 | 5.3 | 7.2 | down | PLL22 |
| At5g48900 | 4.0 | 4.8 |  | PLL21 |
| At3g07010 | 4.0 | 3.8 |  | PLL20 |
| At3g53190 | 7.0 | 6.5 |  | PLL17 |
| At5g09280 | 2.1 | 2.1 |  | PLL3 |
| At1g30350 | 2.5 | 2.6 |  | PLL7 |
| At4g22080 | - | - |  | PLL4 |
| At2g02720 | 2.4 | 2.5 |  | PLL9 |
| At3g54920 | 8.6 | 5.9 | Up | PLL13, PMR6 |
| At1g14420 | 2.8 | 2.7 |  | PLL8 |
| At5g15110 | 2.3 | 2.8 | down | PLL11 |
| At3g01270 | 2.8 | 3.0 |  | PLL10 |
| At3g55140 | 7.4 | 5.9 | up | PLL2 |
| At3g09540 | 3.8 | 6.0 | down | PLL1 |
| At4g22090 | - | - |  | PLL5 |
| At4g24780 | 11.3 | 4.5 | up | PLL19 |
| At5g04310 | 8.9 | 5.2 | up | PLL12 |
| At5g55720 | 2.5 | 2.6 |  | PLL14 |
| At1g11920 | 2.7 | 4.5 | down | PLL6 |

**Arabidopsis polygalacturonases**

Kim, J., Shiu, S.H., Thoma, S., Li, W.H., and Patterson, S.E. (2006). Patterns of expansion and expression divergence in the plant polygalacturonase gene family. *Genome Biol* 7**,** R87.

| Gene | Syncytium | Root | Regulation |
| --- | --- | --- | --- |
|  |  |  |  |
| At2g15450 | - | - |  |
| At2g15470 | - | - |  |
| At2g15460 | - | - |  |
| At2g26620 | 2.4 | 2.6 |  |
| At2g40310 | 2.9 | 3.0 |  |
| At4g13760 | 2.3 | 2.4 |  |
| At1g43080 | - | - |  |
| At1g43090 | - | - |  |
| At1g43100 | - | - |  |
| At1g17150 | 2.9 | 2.9 |  |
| At1g78400 | 2.3 | 2.4 |  |
| At2g33160 | 2.7 | 2.7 |  |
| At1g02790 | 3.0 | 3.2 |  |
| At4g18180 | 3.0 | 3.3 |  |
| At3g07850 | - | - |  |
| At3g14040 | - | - |  |
| At3g07820 | 2.4 | 2.5 |  |
| At3g07840 | 2.4 | 2.5 |  |
| At3g07830 | 3.2 | 3.4 |  |
| At5g48140 | 2.2 | 2.4 |  |
| At2g43860 | 3.3 | 3.4 |  |
| At2g43870 | 2.6 | 2.6 |  |
| At3g59850 | 2.6 | 2.7 |  |
| At1g65570 | 2.6 | 3.0 |  |
| At2g43880 | 2.4 | 2.6 |  |
| At2g43890 | 2.9 | 2.9 |  |
| At1g05650 | - | - |  |
| At1g05660 | - | - |  |
| At1g80140 | 3.1 | 3.1 |  |
| At4g32380 | - | - |  |
| At4g32370 | - | - |  |
| At5g17200 | 2.9 | 2.9 |  |
| At5g39910 | 2.5 | 2.6 |  |
| At3g15720 | 3.2 | 3.3 |  |
| At5g27530 | 3.8 | 4.0 |  |
| At4g35670 | 2.7 | 2.9 |  |
| At5g44830 | 3.0 | 2.9 |  |
| At5g44840 | 3.0 | 2.9 |  |
| At2g41850 | 3.2 | 3.5 |  |
| At3g57510 | 3.4 | 3.5 |  |
| At3g07970 | 2.7 | 3.0 |  |
| At1g80170 | 5.5 | 4.4 | up |
| At1g70500 | 3.0 | 4.2 | down |
| At1g23460 | - | - |  |
| At1g23470 | - | - |  |
| At1g02460 | 4.0 | 4.7 | down |
| At4g01890 | 3.9 | 4.7 |  |
| At1g48100 | 2.3 | 2.5 |  |
| At1g56710 | 2.7 | 2.7 |  |
| At3g26610 | 3.3 | 5.6 | down |
| At5g14650 | 3.0 | 3.6 | down |
| At1g10640 | 2.7 | 2.9 |  |
| At1g60590 | 2.6 | 2.7 |  |
| At1g19170 | 6.1 | 6.1 |  |
| At3g42950 | 3.5 | 3.9 |  |
| At2g23900 | 2.1 | 2.2 |  |
| At3g48950 | 3.8 | 3.8 |  |
| At3g61490 | 5.6 | 5.1 | up |
| At4g23500 | - | - |  |
| At4g23820 | 5.6 | 3.9 | up |
| At5g41870 | 4.2 | 3.6 | up |
| At3g06770 | 4.3 | 5.2 |  |
| At3g16850 | 5.1 | 5.6 |  |
| At3g62110 | 4.8 | 4.3 |  |
| At4g33440 | 5.0 | 4.6 |  |
| At3g57790 | 5.6 | 9.1 | down |

**Arabidopsis pectin methylesterases**

Louvet, R., Cavel, E., Gutierrez, L., Guénin, S., Roger, D., Gillet, F., Guerineau, F. and Pelloux, J. (2006). Comprehensive expression profiling of the pectin methylesterase gene family during silique development in Arabidopsis thaliana. *Planta* 224, 782-91.

| Gene | Syncytium | Root | Regulation | Other names |
| --- | --- | --- | --- | --- |
|  |  |  |  |  |
| At5g51490 | 2.9 | 2.9 |  |  |
| At5g51500 | 2.5 | 3.0 | down |  |
| At3g47400 | 3.0 | 3.7 | down |  |
| At2g45220 | 3.6 | 8.8 | down |  |
| At4g00190 | 2.6 | 2.7 |  |  |
| At1g23200 | 2.9 | 3.2 |  |  |
| At3g60730 | 2.2 | 2.2 |  |  |
| At1g53830 | 3.4 | 3.7 |  |  |
| At3g14310 | 9.4 | 8.9 |  | PME3 |
| At3g05620 | 2.8 | 4.7 | down |  |
| At3g43270 | 4.8 | 4.5 |  |  |
| At4g33220 | 2.3 | 2.4 |  |  |
| At4g02300 | 2.8 | 2.8 |  |  |
| At4g02320 | 2.4 | 2.7 |  |  |
| At2g26440 | 5.7 | 3.7 | Up |  |
| At5g20860 | 2.8 | 2.9 |  |  |
| At2g43050 | 3.1 | 4.5 | down |  |
| At3g59010 | 4.2 | 3.8 |  |  |
| At3g49220 | 7.3 | 6.5 |  |  |
| At5g53370 | 7.3 | 8.7 |  |  |
| At3g10720 | 2.5 | 2.7 |  |  |
| At5g04970 | 2.2 | 2.4 |  |  |
| At2g47550 | 3.7 | 3.1 |  |  |
| At1g02810 | 2.5 | 5.6 | down |  |
| At4g02330 | 4.9 | 5.6 |  |  |
| At1g53840 | 5.4 | 3.3 | up |  |
| At3g14300 | 2.6 | 3.0 |  |  |
| At3g10710 | 4.0 | 6.8 | down |  |
| At5g04960 | 2.9 | 9.8 | down |  |
| At1g11370 | 2.1 | 2.3 |  |  |
| At1g11580 | 10.6 | 9.7 |  |  |
| At1g44980 | 2.6 | 2.7 |  |  |
| At4g03930 | 2.8 | 3.0 |  |  |
| At1g11590 | 2.9 | 2.9 |  |  |
| At3g27980 | 2.0 | 2.1 |  |  |
| At2g26450 | 2.9 | 3.1 |  |  |
| At4g33230 | 3.2 | 3.4 |  |  |
| At2g47030 | - | - |  |  |
| At2g47040 | - | - |  |  |
| At3g62170 | 3.1 | 3.4 |  |  |
| At3g05610 | 2.5 | 2.6 |  |  |
| At5g27870 | 2.7 | 2.9 |  |  |
| At4g15980 | 2.1 | 2.2 |  |  |
| At3g06830 | 3.0 | 3.0 |  |  |
| At5g49180 | 2.5 | 2.6 |  |  |
| At5g09760 | 9.0 | 9.1 |  |  |
| At5g64640 | 4.6 | 3.7 | up |  |
| At2g19150 | 3.3 | 6.6 | down |  |
| At2g47280 | 3.1 | 3.2 |  |  |
| At5g26810 | - | - |  |  |
| At3g24130 | 2.5 | 2.8 |  |  |
| At5g18990 | 2.3 | 2.5 |  |  |
| At2g36700 | 3.2 | 3.3 |  |  |
| At2g36710 | 3.1 | 3.4 |  |  |
| At1g05310 | 4.3 | 5.6 |  |  |
| At5g47500 | 3.1 | 3.2 |  |  |
| At5g55590 | 3.3 | 4.6 |  |  |
| At5g19730 | 4.1 | 8.5 | down |  |
| At3g29090 | 7.5 | 6.6 |  |  |
| At3g17060 | 2.9 | 3.2 |  |  |
| At2g21610 | 2.9 | 3.2 |  |  |
| At5g07420 | 2.4 | 2.4 |  |  |
| At5g07430 | 2.1 | 2.3 |  |  |
| At5g61680 | - | - |  |  |
| At1g69940 | - | - |  |  |
| At5g07410 | - | - |  |  |

**Arabidopsis pectin acetylesterases**

Gou, J.Y., Miller, L.M., Hou, G., Yu, X.H., Chen, X.Y., and Liu, C.J. (2012). Acetylesterase-mediated deacetylation of pectin impairs cell elongation, pollen germination, and plant reproduction. *Plant Cell* 24**,** 50-65.

| Gene | Syncytium | Root | Regulation |
| --- | --- | --- | --- |
|  |  |  |  |
| AT5G23870 | 3.3 | 3.4 |  |
| AT3G09410 | 3.3 | 4.6 | down |
| AT3G09420 | - | - |  |
| AT3G62060 | 5.0 | 3.3 |  |
| AT5G45280 | 5.1 | 5.8 | down |
| AT2G46930 | 7.3 | 6.5 |  |
| AT1G09550 | 2.6 | 2.8 |  |
| AT3G05910 | 8.1 | 5.7 | up |
| AT4G19410 | 11.5 | 9.7 | up |
| AT4G19420 | 3.6 | 4.1 |  |
| AT5G26670 | 5.9 | 4.6 | up |
| AT1G57590 | 3.9 | 3.6 |  |
